# Humoral and cellular immunogenicity of COVID-19 booster dose vaccination in inflammatory arthritis patients

**DOI:** 10.3389/fimmu.2022.1033804

**Published:** 2022-10-31

**Authors:** Jakub Wroński, Bożena Jaszczyk, Leszek Roszkowski, Anna Felis-Giemza, Krzysztof Bonek, Anna Kornatka, Magdalena Plebańczyk, Tomasz Burakowski, Barbara Lisowska, Brygida Kwiatkowska, Włodzimierz Maśliński, Małgorzata Wisłowska, Magdalena Massalska, Marzena Ciechomska, Ewa Kuca-Warnawin

**Affiliations:** ^1^ Department of Rheumatology, National Institute of Geriatrics, Rheumatology and Rehabilitation, Warsaw, Poland; ^2^ Department of Outpatient Clinics, National Institute of Geriatrics, Rheumatology and Rehabilitation, Warsaw, Poland; ^3^ Biologic Therapy Center, National Institute of Geriatrics, Rheumatology and Rehabilitation, Warsaw, Poland; ^4^ Department of Pathophysiology and Immunology, National Institute of Geriatrics, Rheumatology and Rehabilitation, Warsaw, Poland; ^5^ Department of Anesthesiology, National Institute of Geriatrics, Rheumatology and Rehabilitation, Warsaw, Poland; ^6^ Department of Early Arthritis, National Institute of Geriatrics, Rheumatology and Rehabilitation, Warsaw, Poland

**Keywords:** COVID-19, booster vaccine, arthritis, immunogenicity, cellular response, humoral response

## Abstract

**Introduction:**

Previous studies have shown a reduction in the effectiveness of primary COVID-19 vaccination in patients with rheumatic diseases. However, limited data is available regarding the effectiveness of the COVID-19 vaccine booster dose, especially on cellular response. The study aimed to assess the humoral and cellular immunogenicity of a booster dose in patients with inflammatory arthritis (IA).

**Patients and methods:**

49 IA and 47 age and sex-matched healthy controls (HC) were included in a prospective cohort study. Both groups completed primary COVID-19 vaccination and after more than 180 days received a BNT162b2 booster shot. Humoral responses (level of IgG antibodies) and cellular responses (IFN-γ production) were assessed before and after 4 weeks from the booster dose of the vaccine.

**Results:**

After the booster dose, all participants showed an increased humoral response, although significantly reduced antibody levels were observed in IA patients compared to HC (p=0.004). The cellular response was significantly lower both before (p<0.001) and after (p<0.001) the booster dose in IA patients as compared to HC. Among the immunomodulatory drugs, only biological and targeted synthetic drugs lowered the humoral response after booster vaccination. However, the cellular response was decreased after all immunomodulatory drugs except IL-17 inhibitors and sulfasalazine.

**Conclusion:**

Our data indicate that patients with rheumatic diseases present lower humoral and cellular responses after the COVID-19 booster vaccine in comparison to HC. This may translate into a recommendation for subsequent booster doses of the COVID-19 vaccine for rheumatic patients.

## 1 Introduction

Patients treated with immunosuppressive drugs have an increased risk of developing COVID-19. Among the more vulnerable patients, there are patients with autoimmune inflammatory rheumatic diseases (AIIRD) (1–3). Data on the course of COVID-19 in AIIRD patients is more ambiguous. Despite the lack of risk of more frequent hospitalizations or intensive care unit admissions (2, 3), a recent meta-analysis showed a slight increase in mortality in AIIRD patients compared to the general population (3). COVID-19 vaccines have not fully resolved the problem of patients undergoing immunomodulatory therapy, as such therapy may result in reduced vaccine response. Numerous studies showed that despite the full vaccination regimen, patients with AIIRD are at increased risk of lack of humoral response (4–10), and among AIIRD patients who have developed post-vaccine antibodies their levels are lower than in healthy people (10–12). Immunomodulatory drugs that reduce the immunogenicity of COVID-19 vaccines to the greatest extent are rituximab (RTX) (4, 5, 8–10, 12–16), mycophenolate mofetil (MMF) (5, 8–10, 13, 15, 17), and abatacept (ABT) (8, 9, 18). A similar immunogenicity-lowering effect was shown for glucocorticosteroids (GCs) (5, 7, 9, 10, 13, 15). Data on other immunomodulatory drugs are contradictory, although single studies are showing a decrease in the humoral response after methotrexate (MTX) (6, 7, 9, 17) and IL-6 inhibitors (18). A weaker humoral response may, in turn, contribute to a premature decline in post-vaccination immunity, especially in patients treated with RTX (19), GCs (19), TNF inhibitors (11, 20), and ABT (20). The humoral response is only one of the components of the vaccine response. The cellular response was recorded in patients who did not show a humoral response after RTX (14, 16, 21, 22). Still, the exact role of the cellular response in ensuring the effectiveness of COVID-19 vaccination remains unknown.

This naturally raises the question of the need and the optimal timing for a COVID-19 additional vaccine dose for AIIRD patients. Initially, following studies on other groups of immunocompromised patients (i.e. solid-organ transplant recipients (23)), research attempts were made to modify the primary vaccination schedule in AIIRD patients by giving additional vaccine doses shortly after the standard schedule (16, 24, 25). Currently, research is focused on studying the immunogenicity of the COVID-19 booster doses – additional doses given to patients after their response may have waned over time (26). Unfortunately, despite the commencement of widespread COVID-19 vaccination with booster doses in many countries around the world, data on the effectiveness of COVID-19 vaccine booster doses in patients with AIIRD is scarce (27, 28). There are only a few studies describing the immunogenicity of a booster dose in AIIRD patients (29–33), but no study evaluating individual immunomodulatory drugs’ effect on cellular response to booster dose in AIIRD patients. The aim of our study was therefore to assess the immunogenicity of a booster dose of COVID-19 vaccination on humoral and cellular levels in inflammatory arthritis (IA) cohort treated with immunomodulatory drugs.

## 2 Patients and methods

### 2.1 Patients

The study was conducted at the COVID-19 vaccination center in a rheumatology center. IA patients and healthy controls (HC; sex and age-matched) visiting the vaccination center between November 2021 to January 2022 were enrolled. The inclusion criteria for both groups included age above 18, willingness to get vaccinated with a booster dose of the COVID-19 vaccination (BNT162b2, Pfizer-BioNTech), and a period longer than 6 months from the end of primary COVID-19 vaccination. The additional inclusion criterion for the IA group was a diagnosis of rheumatoid arthritis (RA) according to the ACR-EULAR 2010 criteria, ankylosing spondylitis (AS) according to modified New York criteria, psoriatic arthritis (PsA) according to CASPAR criteria, or non-radiographic spondyloarthritis (nrSpA) according to ASAS 2010 criteria. The exclusion criteria for both groups were a previous allergic reaction to vaccination against COVID-19, serious adverse event (SAE) after previous vaccination against COVID-19, or other conditions which, in the opinion of the qualifying physician, constitute a contraindication to vaccination. The additional exclusion criterion in the control group was treatment with any kind of immunomodulatory therapy. The study protocol was approved by the hospital bioethics committee (KBT-3/2/2021). All participants signed informed consent for inclusion in the study. The study was conducted according to the Declaration of Helsinki.

### 2.2 Methods

Patient characteristics (including use of drugs before the booster vaccination and during the primary vaccination schedule) and data on vaccination safety, including the occurrence of adverse events (AE) and their grading according to Common Terminology Criteria for Adverse Events v5.0 (CTCAE), were collected by qualifying physicians using a structured interview. Data regarding primary COVID-19 vaccinations and previous COVID-19 infections were gathered from both interviews and the national COVID-19 registry. Additionally, to detect previous asymptomatic COVID-19 infection, SARS-CoV N ELISA Kit (TestLine Clinical Diagnostics, Brno, Czech Republic) detecting antibodies against SARS-CoV-2 nucleocapsid was performed. Blood samples were collected from all recruited patients before the booster COVID-19 vaccination and 4 weeks after the booster vaccination. Data regarding patient characteristics was blinded to the laboratory staff.

#### 2.2.1 Humoral immunity assessment

To determine the concentration of IgG antibodies against the SARS-CoV-2 S1 antigen, anti-SARS-CoV-2 QuantiVac ELISA (Euroimmun, Lübeck, Germany) test was used. A cut-off value of 8 RU/mL was used to define positive test values according to the manufacturer’s instructions.

#### 2.2.2 Cellular immunity assessment

The cellular response was measured in only part of the patients, maintaining sex and age matching (the exact number of patients tested with each method is shown in the Results section, **Figures 2**, **3**).

##### 2.2.2.1 SARS-CoV-2 interferon gamma release assay (IGRA)

To evaluate cellular response against viral proteins Quan-T-Cell SARS-CoV-2 (Euroimmun, Lübeck, Germany) test was used. A cut-off value of 18.44 mIU/mL was used to define positive test values according to the manufacturer’s instructions. In the first stage, freshly drawn heparinized whole blood was incubated with the S1 antigen of the SARS-CoV-2 virus coated on the bottom of the test tube. Whole blood was also incubated in a second negative control tube (assessment of non-specific background response) and a third positive control tube (assessment of overall T cell response after stimulation). After incubation time (22-24h), serum plasma was obtained. In the second stage, an ELISA test was performed to measure the secreted IFN-γ in the first step of the test.

##### 2.2.2.2 Cell isolation and stimulation

Peripheral blood mononuclear cells (PBMC) were isolated from heparinized blood by density gradient centrifugation using Ficoll-Paque (GE Healthcare Bio-Sciences, Uppsala, Sweden). 2,5 × 105 PBMC/well were cultured in 96-well plates (Nunc, Thermo Fisher Scientific, Waltham, MA, USA) in 200μl RPMI 1640 medium (Invitrogen, Paisley, UK) supplemented with 10% heat-inactivated fetal calf serum (FCS) (Biochrom AG, Berlin, Germany), 100 U/ml penicillin, 100 μg/ml streptomycin (both antibiotics from Polfa Tarchomin, Warsaw, Poland), 30 μg/ml kanamycin (Sigma, St Louis, MO, USA) and 1 mM HEPES (Invitrogen) for 72 hours. At the beginning of the culture, cells were stimulated with viral variants with PepMix™ SARS-CoV-2 (S-RBD B.1.617.2/Delta) (JPT Peptide Technologies, Berlin Germany) or recombinant SARS-CoV-2 Spike S1+S2 (R683A, R685A) Trimer (BioLegend, San Diego, CA, USA).

We used Delta protein because this variant was dominant in the Polish population during the blood collection. Each protein was used at a concentration of 1μg/mL. Unstimulated cells cultured without viral proteins served as a negative control.

##### 2.2.2.3 Flow cytometry

The cells were washed and stained for surface antigens using anti-CD3 FITC, antibody. Subsequently, cells were fixed and permeabilized using BD Cytofix/Cytoperm kit. Intracellular stainings using anti-IFN-γ-PE and appropriate isotype control antibodies were done. After the washing step, cells were acquired and analyzed using a FACSCanto II cell sorter/cytometer and Diva software. All used reagents were purchased from Becton Dickinson (San Jose, CA, USA). In stimulation experiments, frequencies of activated CD3+ IFN-γ+, T cells were background-subtracted, with the frequency in the negative (unstimulated) control sample representing the background. The representative gating strategy is shown in **Supplementary Figure 1**.

### 2.3 Statistics

The compliance of the data with the normal distribution was assessed using the Shapiro–Wilk test. The significance of the observed differences between the two groups was assessed using the Student’s t-test for variables with a normal distribution, the Mann–Whitney U test for variables without a normal distribution, and for categorical variables, the Chi-square test or the Fisher’s exact test (for tables with values less than 5). For more than two groups without normal distribution, we used the Kruskal–Wallis test with *post hoc* analysis with the Dunn’s test. The correlation was assessed using Spearman’s rank correlation coefficient with non-parametric variables. The effect of mitogen stimulation was assessed by the Wilcoxon test. The significance of the correlation after adjusting for the confounding factors was checked by linear regression. The multivariant ANOVA analysis was performed to identify the predictors of a higher AE rate. In both multivariant analyses, only patients without missing data were included. Statistical significance was set at p < 0.05. Statistical analysis was performed using Statistica 13.3 software (StatSoft Polska, Cracow, Poland). Figures were created using GraphPad Prism 6 software (GraphPad Software, San Diego, CA, US).

## 3 Results

### 3.1 Patient characteristics

The study involved 96 patients – 49 with IA and 47 HC. Patient characteristics are presented in **Table 1**. There were no significant differences between the groups, apart from the greater number of ex-smokers in the IA group and the longer interval between the primary vaccinations and the booster dose in the HC group. The types of immunomodulatory treatment received and patients’ diseases among the IA group are presented in **Table 2**.

**Table 1:** Patient characteristics.

	Inflammatory arthritis (n=49)	Healthy control (n=47)	difference
Age (mean ± SD)	53 ± 13.9	48.6 ± 14.1	ns
Sex – female (n, %)	34 (68%)	33 (71.7%)	ns
BMI (median, min, max)	26.2 (17.5, 41.6)	25.2 (18, 43.6)	ns
Smoking (n, %)			
-current -past	5 (10.2%) 13 (26.5%)	4 (8.5%) 4 (8.5%)	ns p=0.031
Days after the end of primary vaccination (median, min, max)	196 (123, 314)	267 (105, 355)	p<0.001
Days after booster vaccination (median, min, max)	31 (22, 52)	31 (27, 77)	ns
COVID-19 infection before booster vaccination (n, %) -asymptomatic	14 (28.6%) 4 (8.2%)	18 (38.3%) 7 (14.9%)	ns ns
Vaccine primary scheme (n, %)			
-Pfizer -AstraZeneca -Moderna -Johnson&Johnson -Unknown	37 (75.5%) 6 (12.2%) 4 (8.2%) 2 (4.1%) -	41 (87.2%) 4 (8.5%) 1 (2.1%) - 1 (2.1%)	ns
Vaccine booster dose (n, %)			
-Pfizer	49 (100%)	47 (100%)	ns
COVID-19 infection after booster vaccination (n, %)	7 (14.3%)	12 (25.5%)	ns

ns, non significant.

**Table 2:** Inflammatory arthritis group characteristics.

Disease type (n, %)	
-Rheumatoid arthritis -Ankylosing spondylitis -Psoriatic arthritis -nrSpA	28 (57.1%) 12 (24.5%) 8 (16.3%) 1 (2%)
**Immunomodulatory treatment during primary vaccination (n=43, 87.7%)**	
GCs (n, %)	11 (22.4%)
-dose (median, min, max)	5 (2.5, 10)
cDMARDs (n, %)	
-MTX -SSA -HCQ	21 (42.8%) 5 (10.2%) 1 (2%)
bDMARDs and tsDMARDs (n, %)	28 (57.1%)
-TNFi -IL-6i -JAKi -IL-17i	15 (30.6%) 5 (10.2%) 5 (10.2%) 3 (6.1%)
bDMARDs and tsDMARDs used:	
-in monotherapy -with cDMARDs	16 (37.2%) 12 (27.9%)
Treatment suspension during primary vaccination (n, %)	8 (16.3%)
**Immunomodulatory treatment during booster vaccination (n=46, 93.9%)**	
GCs (n, %)	14 (28.6%)
-dose (median, min, max)	5 (2.5, 37.5)
cDMARDs (n, %)	
-MTX -SSA -HCQ	24 (49%) 6 (12.2%) 1 (2%)
bDMARDs and tsDMARDs (n, %)	31 (63.3%)
-TNFi -JAKi -IL-6i -IL-17i	17 (34.7%) 7 (14.3%) 5 (10.2%) 3 (6.1%)
bDMARDs and tsDMARDs used:	
-in monotherapy -with cDMARDs	19 (41.3%) 12 (26.1%)
Treatment suspension during booster vaccination (n, %)	3 (6.1%)

bDMARDs, biological disease-modifying antirheumatic drugs; GCs, glucocorticoids; HCQ, hydroxychloroquine; IL-6i, IL-6 inhibitors; IL-17i, IL-17 inhibitors; JAKi, JAK inhibitors; MTX, methotrexate; nrSpA, non-radiographic spondylarthritis; ns, nonsignificant; SSA, sulfasalazine; TNFi, TNF inhibitors; tsDMARDs, targeted synthetic disease-modifying antirheumatic drugs.

### 3.2 Humoral responses to SARS-CoV-2 vaccination in IA patients

**Figure 1:**
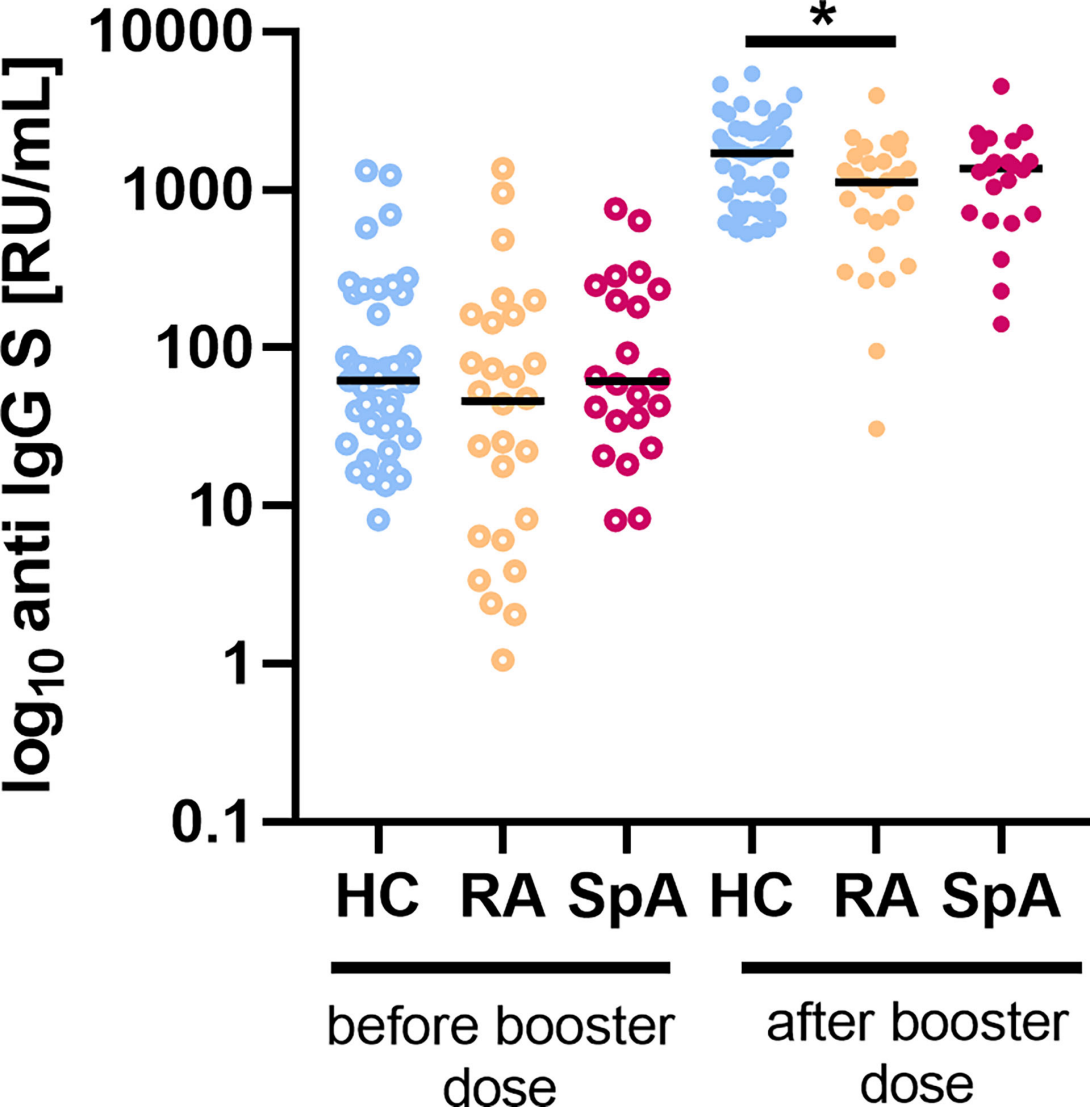
The level of IgG anti-S before and after a booster dose of the COVID-19 vaccine in patients with rheumatoid arthritis (RA; n=28) and spondyloarthritis (SpA; n=21) compared to healthy controls (HC; n=43/47 before/after). In the group comparison, the Kruskal–Wallis test with *post hoc* analysis with the Dunn’s test was performed. Dots represent individual values and the line represents the median. A p values were expressed as follows: 0.05>p>0.01 as*.

Both HC and IA patients before a booster dose of the COVID-19 vaccine showed low levels of anti-S protein IgG antibodies (**Figure 1**). The study showed a reduced persistence of the humoral response among patients in the IA group (79.6%) after 6 months from primary vaccination compared to HC (100%, p<0.001). After a booster dose, levels of antibodies raised in both HC and IA, but significantly more in HC (median 1693 RU/ml) compared to all IA patients (median 1227 RU/ml, p=0.004), with the lowest levels of anti-S protein IgG antibodies in RA patients (median 1040 RU/ml, p=0.016). In IA patients, we did not observe any correlation between antibody levels before and after the booster. However, in HC such a correlation exists (r=0.38, p=0.01; **Supplementary Figure 2**), which may indicate impairment of humoral immunity in IA patients. The kinetics of anti-S-IgG concentration are shown in **Supplementary Figure 3**.

### 3.3 Cellular responses after SARS-CoV-2 vaccination in IA patients

**Figure 2:**
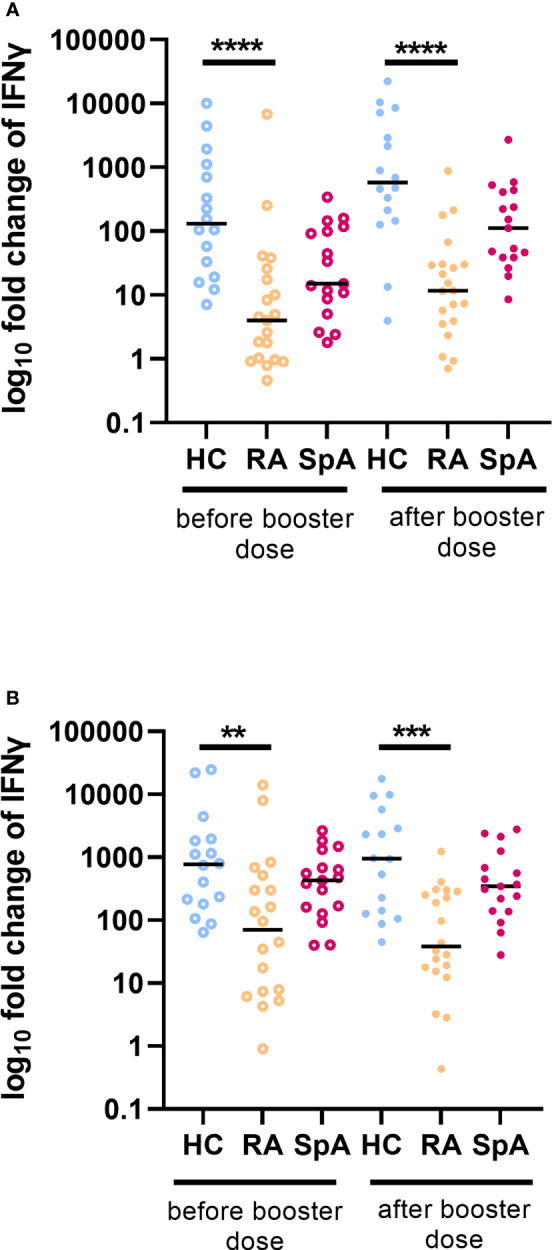
Fold change of INF-γ production after **(A)** viral protein and **(B)** mitogen stimulation in patients with rheumatoid arthritis (RA; n=20) and spondyloarthritis (SpA; n=25) compared to healthy controls (HC; n=20) before and after a booster dose of COVID-19 vaccine. In the group comparison, the Kruskal–Wallis test with *post hoc* analysis with the Dunn’s test was performed. Dots represent individual values and the line represents the median. A p values were expressed as follows: 0.01>p>0.001 as**; p<0.001 as***; p<0.0001 as****.

The IFN-γ production in the whole blood after viral protein stimulation was significantly lower in the IA group (median 9.4 fold change) in comparison to the HC (median 130.8 fold change; p<0.001) before a booster vaccination. After the booster dose, similarly lower IFN-γ production in the IA group (median 30.3 fold change) compared to the HC (median 580.6 fold change; p<0.001) was shown. Analyzing the individual types of IA, significantly lower IFN-γ production after viral protein stimulation was observed in the RA patients group as compared to HC both before and after the booster dose (**Figure 2**). Stimulation by mitogen has resulted in higher IFN-γ production than stimulation by viral proteins. The mitogen-stimulated IFN-γ production was significantly lower in RA in comparison to HC before and after the booster dose. The kinetics of viral antigen-stimulated fold change of IFN is shown in **Supplementary Figure 4**.

**Figure 3:**
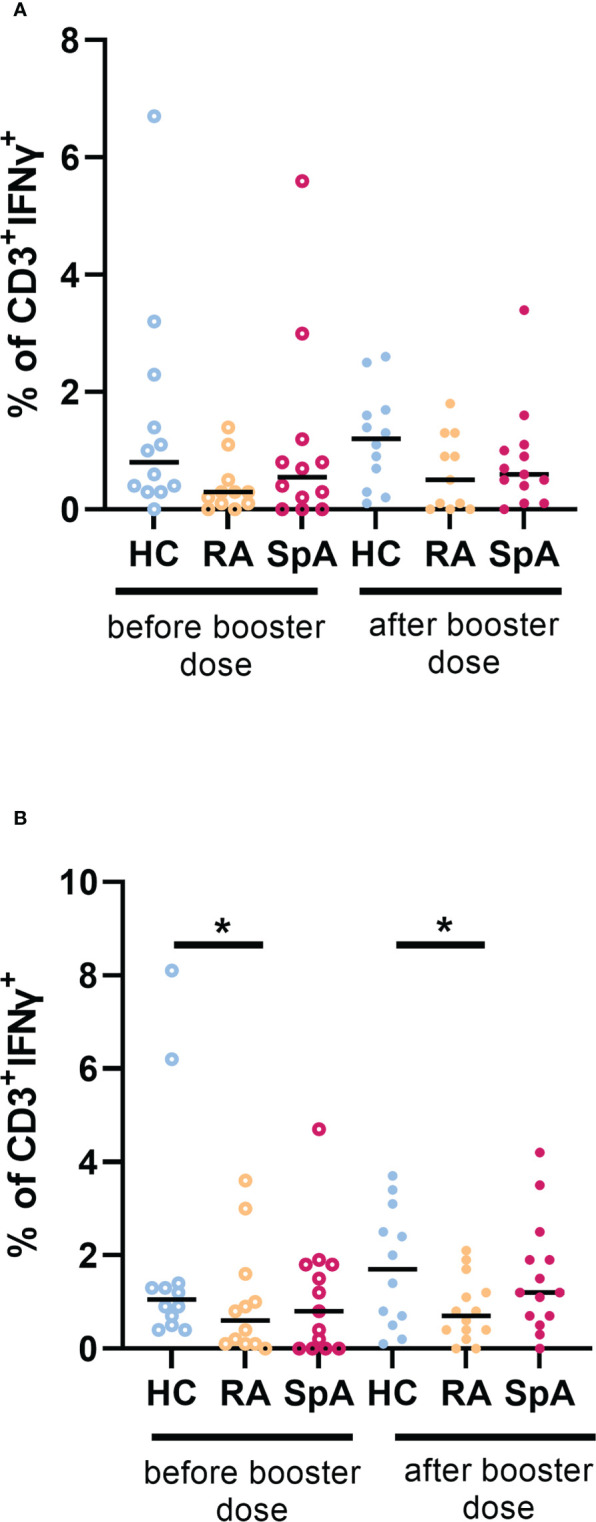
**(A)** Percentage of CD3+ INF-γ+ cells after wild-type viral protein stimulation in patients with rheumatoid arthritis (RA; n=10) and spondyloarthritis (SpA; n=14) compared to healthy controls (HC; n=12) before and after a booster dose of COVID-19 vaccine. **(B)** Percentage of CD3+ INF-γ+ cells after delta viral protein stimulation in patients with RA (n=11) and SpA (n=15) compared to HC (n=11) before and after booster dose of COVID-19 vaccine. In the group comparison, the Kruskal–Wallis test with *post hoc* analysis with the Dunn’s test was performed. Dots represent individual values and the line represents the median. A p values were expressed as follows: 0.05>p>0.01 as*.

The cellular response was also assessed as intracellular IFN-γ production by T cells. Both stimulators: virus wild-type protein and Delta protein increased the production of IFN-γ by T cells. The percentage of T cells secreting IFN-γ after stimulation with virus wild-type protein was significantly lower in the IA group (median 0.6 fold change) compared to HC (median 1.2 fold change) after the booster dose of vaccination. Such a difference was not observed before the booster dose. Considering diagnosis, we observed that the group of RA patients showed significantly lower intracellular production of IFN-γ both before and after the booster dose of vaccination compared to the HC (**Figure 3**). However, we did not observe any differences between IA patients and HC in intracellular IFN-γ production after Delta protein stimulation. Interestingly, the cellular response to the Delta variant was maintained in 100% of HC and 93% of IA patients. The kinetics of the percentage of CD3+ INF-γ+ cells after wild-type viral protein stimulation is shown in **Supplementary Figure 5**.

### 3.4 Effect of immunomodulatory drugs on the immunogenicity of the COVID-19 vaccines

The analysis of the effect of immunomodulatory drugs on the immunogenicity of the COVID-19 vaccines is shown in **Table 3** (A – before the booster dose, B – after the booster dose). The analysis showed a significantly reduced cellular response compared to the control group 6 months after primary vaccination with all drugs except JAK and IL-17 inhibitors. Similarly, all drugs except IL-17 inhibitors and sulfasalazine reduced cellular response 4 weeks after the booster dose. The humoral response was lowered after primary vaccination by IL-6 inhibitors (p=0.044), and after booster dose by JAK inhibitors (p=0.004), IL-6 inhibitors (p<0.001), and all biological drugs combined (p=0.001). There were no statistically significant differences in humoral and cellular responses between patients using biological and targeted synthetic disease-modifying antirheumatic drugs in monotherapy vs in combination with conventional disease-modifying antirheumatic drugs, both before and after booster vaccination.

**Table 3A:** Effect of immunomodulating drugs on the immunogenicity of the COVID-19 vaccines more than 6 months from primary vaccination.

IgG antibodies, median RU/ml (min, max)				
Healthy control (HC, n=43)	62.2 (8.2, 1326.9)		Difference compared to HC	
GCs (n=11)	22.3 (1.1, 1364.9)		ns	
MTX (n=21)	65.7 (2.1, 303.9)		ns	
SSZ (n=5)	8.3 (3.4, 200.1)		ns	
bDMARDs and tsDMARDs (n=28)	43.8 (0, 953.8)		ns	
- TNFi (n=15)	42.4 (3.9, 637.9)		ns	
- IL-6i (n=5)	6.1 (1.1, 145)		p=0.044	
- JAKi (n=5)	80 (0, 953.8)		ns	
- IL-17i (n=3)	236.8 (181.9, 250.2)		ns	
**IGRA, median fold change (min, max)**				
Healthy control (HC, n=16)	130.8 (7.1, 10015.3)		Difference compared to HC	
GCs (n=9)	1 (0.5, 252.3)		p=0.004	
MTX (n=16)	6.7 (0.5, 159.5)		p<0.001	
SSZ (n=3)	1.8 (0.9, 38.3)		p=0.033	
bDMARDs and tsDMARDs (n=20)	7.5 (0, 6823.6)		p=0.001	
- TNFi (n=8)	8.4 (1.8, 159.5)		p=0.006	
- IL-6i (n=5)	1 (0.5, 40.7)		p=0.002	
- JAKi (n=4)	5.5 (0, 6823.6)		ns	
- IL-17i (n=3)	91.6 (44.5, 145.6)		ns	
**% of CD3+INFγ+ after Delta (median, min, max)**				
Healthy control (HC, n=12)	1.1 (0.4, 8.1)		Difference compared to HC	
GCs (n=4)	0.1 (0, 0.4)		p=0.002	
MTX (n=10)	0.5 (0, 3)		ns	
SSZ (n=2)	0.1 (0.1, 0.1)		p=0.022	
bDMARDs and tsDMARDs (n=15)	0.8 (0, 3.6)		ns	
- TNFi (n=8)	0.3 (0, 1.9)		ns	
- IL-6i (n=4)	0.9 (0, 1.6)		ns	
- JAKi (n=2)	1.9 (0.1, 3.6)		ns	
- IL-17i (n=1)	1.8 (1.8, 1.8)		–	
**% of CD3+INFγ+ after Wild type, fold change (median, min, max)**				
Healthy control (HC, n=12)	1.7 (0.1, 3.7)		Difference compared to HC	
GCs (n=6)	0.4 (0, 2.1)		ns	
MTX (n=11)	0.5 (0, 1.9)		p=0.012	
SSZ (n=2)	0.6 (0.4, 0.8)		–	
bDMARDs and tsDMARDs (n=17)	1.1 (0, 4.2)		ns	
- TNFi (n=8)	1.6 (0, 4.2)		ns	
- IL-6i (n=5)	0.6 (0, 1.2)		ns	
- JAKi (n=2)	0.4 (0.4, 0.4)		–	
- IL-17i (n=2)	1.1 (0.3, 1.9)		–	

bDMARDs, biological disease-modifying antirheumatic drugs; GCs, glucocorticoids; IL-6i, IL-6 inhibitors; IL-17i, IL-17 inhibitors; JAKi, JAK inhibitors; MTX, methotrexate; ns, nonsignificant; SSZ, sulfasalazine; TNFi, TNF inhibitors; tsDMARDs, targeted synthetic disease-modifying antirheumatic drugs.

**Table 3B:** Effect of immunomodulating drugs on the immunogenicity of the COVID-19 vaccines after 4 weeks from booster dose.

IgG antibodies, median RU/ml (min, max)			
Healthy control (HC, n=47)	1693.1 (530.5, 5419.1)	Difference compared to HC	
GCs (n=14)	1167.7 (30.8, 4524.7)	ns	
MTX (n=24)	1199.4 (30.8, 4724.7)	ns	
SSZ (n=6)	1311.2 (30.8, 3950.7)	ns	
bDMARDs and tsDMARDs (n=31)	999.1 (95.5, 2325.4)	p=0.001	
- TNFi (n=17)	1304.5 (141.6, 2142.2)	ns	
- IL-6i (n=5)	329.8 (272.2, 999.1)	p=0.004	
- JAKi (n=7)	614.8 (95.5, 1870.9)	p<0.001	
- IL-17i (n=3)	2295.8 (1504.4, 2325.4)	ns	
**IGRA, median fold change (min, max)**			
Healthy control (HC, n=16)	580.6 (4, 22303.4)	Difference compared to HC	
GCs (n=12)	7.7 (0.7, 213)	p<0.001	
MTX (n=20)	34.6 (0.7, 2699.7)	p<0.001	
SSZ (n=4)	60.3 (5.7, 527.8)	ns	
bDMARDs and tsDMARDs (n=23)	30.4 (0.7, 2699.7)	p<0.001	
- TNFi (n=10)	75.4 (8.5, 2699.7)	p=0.047	
- IL-6i (n=5)	7.1 (0.7, 179.7)	p<0.001	
- JAKi (n=6)	11.6 (2.3, 21.4)	p=0.004	
- IL-17i (n=3)	151.5 (48.4, 409.5)	ns	
**% of CD3+INFγ+ after Delta (median, min, max)**			
Healthy control (HC, n=12)	1.7 (0.1, 3.7)	Difference compared to HC	
GCs (n=8)	0.4 (0, 2.1)	ns	
MTX (n=13)	0.8 (0, 2.1)	ns	
SSZ (n=2)	1 (0.8, 1.2)	ns	
bDMARDs and tsDMARDs (n=19)	1.1 (0, 4.2)	ns	
- TNFi (n=9)	1.7 (0, 4.2)	ns	
- IL-6i (n=5)	0.6 (0, 1.2)	ns	
- JAKi (n=3)	0.4 (0.4, 0.5)	ns	
- IL-17i (n=2)	1.1 (0.3, 1.9)	ns	
**% of CD3+INFγ+ after Wild type, fold change (median, min, max)**			
Healthy control (HC, n=12)	1.2 (0.1, 2.6)	Difference compared to HC	
GCs (n=6)	0.7 (0, 1.8)	ns	
MTX (n=11)	0.5 (0, 1.3)	p=0.037	
SSZ (n=1)	0.1 (0.1, 0.1)	–	
bDMARDs and tsDMARDs (n=18)	0.5 (0, 3.4)	ns	
- TNFi (n=9)	1 (0, 3.4)	ns	
- IL-6i (n=5)	0.1 (0, 0.9)	ns	
- JAKi (n=2)	0.3 (0.1, 0.5)	p=0.019	
- IL-17i (n=2)	0.8 (0.4, 1.1)	ns	

bDMARDs, biological disease-modifying antirheumatic drugs; GCs, glucocorticoids; IL-6i, IL-6 inhibitors; IL-17i, IL-17 inhibitors; JAKi, JAK inhibitors; MTX, methotrexate; ns, nonsignificant; SSZ, sulfasalazine; TNFi, TNF inhibitors; tsDMARDs, targeted synthetic disease-modifying antirheumatic drugs.

### 3.5 Effect of other factors on the immunogenicity of the COVID-19 vaccines

The study showed no significant correlation of immunogenicity levels with the interval between the primary immunization and the booster dose. Age of the patients correlated negatively with some results of the cellular response – IFN-γ production by CD3^+^ cells after stimulation with wild-type proteins measured after 6 months (R=-0.4, p=0.014) and IGRA after booster dose (R=-0.3, p=0.025). However, age’s effect on the humoral response was not confirmed. After 6 months from primary vaccination patients who had COVID-19 infection before the primary vaccination had higher antibody levels (p<0.001) and higher cellular response (measured by IGRA, p=0.005) compared to those who did not have COVID-19. Still, prior COVID-19 infection did not affect the immunogenicity of a booster vaccination. Another factor affecting the immunogenicity of the vaccine was active smoking. Patients with active smoking had lower antibody titers after 6 months (p=0.014). Past smoking remained irrelevant. The type of vaccine in the baseline regimen also did not affect the levels of humoral and cellular responses.

### 3.6 Safety of COVID-19 vaccines among patients with IA

The study also assessed the safety of COVID-19 vaccines among patients with IA. The incidence of AE after primary vaccinations is presented in **Table 4**. Although half of the subjects experienced AE, most of the AE were mild, there were only isolated cases of CTCAE grade 3 AE. The frequency of AE did not differ among the patients with IA and the HC. People experiencing AE were younger (p=0.013), had higher antibody levels (both after 6 months from primary vaccination p=0.015 and after a booster dose p=0.031), as well as a better cellular response after booster dose (p=0.038). After accounting for the interaction between age and immune response levels, a significant relationship remained between the incidence of AE and higher antibody levels after 6 months from primary vaccination (p=0.027).

**Table 4:** Adverse events (AE) after primary vaccination in inflammatory arthritis patients and healthy controls.

Number (%)	All patients (n=96)	Inflammatory arthritis (n=49)	Healthy control (n=47)	difference
Any AE	48 (50%)	26 (53.1%)	22 (46.8%)	ns
Injection site reaction -mild -moderate	36 (37.5%) 29 (30.2%) 7 (7.3%)	18 (36.7%) 14 (28.6%) 4 (8.2%)	18 (38.3%) 15 (31.9%) 3 (6.4%)	ns
Malaise -mild -moderate -severe	28 (29.2%) 20 (20.1%) 7 (7.3%) 1 (1%)	16 (32.7%) 12 (24.5%) 3 (6.1%) 1 (2%)	12 (25.5%) 8 (17%) 4 (8.5%) -	ns
Myalgia -mild -moderate -severe	28 (29.2%) 21 (21.9%) 6 (6.25%) 1 (1%)	16 (32.7%) 10 (20.4%) 5 (10.2%) 1 (2%)	12 (25.5%) 11 (23.4%) 1 (2.1%) -	ns
Fever -mild -moderate	22 (22.9%) 17 (17.7%) 5 (5.2%)	15 (30.6%) 11 (22.4%) 4 (8.2%)	7 (14.9%) 6 (12.8%) 1 (2.1%)	ns
Headaches -mild -moderate -severe	16 (16.7%) 12 (12.5%) 3 (3.1%) 1 (1%)	9 (18.4%) 6 (12.2%) 2 (4.1%) 1 (2%)	7 (14.9%) 6 (12.8%) 1 (2.1%) -	ns
Arthralgia -mild -moderate -severe	12 (12.5%) 9 (9.4%) 2 (2.1%) 1 (1%)	7 (14.3%) 5 (10.2%) 1 (2%) 1 (2%)	5 (10.6%) 4 (8.5%) 1 (2.1%) -	ns
Sleep disturbance, mild	2 (2.1%)	2 (4.1%)	–	ns
Syncope, moderate	1 (1%)	1 (2%)	–	ns
Allergic reaction, mild	1 (1%)	1 (2%)	–	ns

ns, nonsignificant.

## 4 Discussion

In our study, we assessed the immunogenicity of COVID-19 booster vaccination in IA patients, including the effect of individual immunomodulatory drugs on both humoral and cellular immunity. There was no difference in the median IgG anti-S levels between the IA group and HC before the booster dose. Still, our study showed a lack of humoral response in part of the IA group 6 months after primary vaccination (in 20.4% of IA patients versus none in HC). This effect was most pronounced in patients treated with IL-6 inhibitors. We demonstrated also a significantly lower IFN-γ production in IA patients as compared to HC before the booster dose of the vaccine, lowered by all immunomodulatory drugs except JAK and IL-17 inhibitors. Our study showed an increase in the humoral response after a booster dose, but the obtained antibody levels in IA patients were lower compared to the HC. The lowest levels of antibodies were obtained in patients treated with IL-6 and JAK inhibitors. Compared to the HC, the cellular response after the booster vaccine was reduced in IA patients treated with most of the immunomodulatory drugs except IL-17 inhibitors and sulfasalazine. The study also assessed the safety of vaccination against COVID-19 in patients with IA. Although most IA patients (53%) experienced AE, the majority were mild AE, without SAE.

According to current EULAR (34) and ACR (26) guidelines, vaccination with a booster dose is recommended in patients with AIIRD. Though in our study there was no difference in the median IgG anti-S levels between the IA group and HC more than 6 months after primary vaccination (most likely due to the small sample size), previous studies have shown a gradual reduction and loss of the humoral response in patients with AIIRD over time (11, 19, 20, 30). However, so far only a few studies on the humoral response after the COVID-19 vaccine booster have been published. Our data is in line with the study by Le Moine et al., who showed a significant increase in SARS-CoV-2 spike-specific antibody levels 6 months after primary vaccination after the booster dose in patients with RA compared with RA patients who did not receive a booster dose (31). A similar increase, in IgG-neutralizing antibody levels, was shown after the booster dose in the study by Ferri et al., lower in AIIRD patients than in healthy controls (32). The study by Benucci et al. showed increased antibody levels after the COVID-19 vaccine booster dose in RA patients but lowered humoral response after ABT and RTX (29). Similarly, the study by Connolly et al. observed an increase in antibody titers after a booster dose and lowered response in patients treated with RTX and MMF (30). Our study confirmed an increase in the humoral response after a booster dose, but the obtained antibody levels were lower compared to the HC, which may suggest a faster decline of the response and the need for further booster doses in the future.

COVID-19 vaccines are also able to induce a cell-mediated immune response. The current research suggests that in AIIRD patients, the efficacy of cellular response may be also diminished after the primary vaccination in comparison to the general population (16, 17, 20). Up to date, only one study evaluated both humoral and cellular responses after booster vaccination in AIIRD patients. Assawasaksakul et al. assessed patients with systemic lupus erythematosus and RA and showed an increase in both humoral and cellular responses after the booster dose (33). The authors however did not compare it with a control group. In our study cellular response appeared to be reduced in IA patients treated with most of the immunomodulatory drugs as compared to the HC. These results may indicate that the cellular response may be more sensitive to immunomodulatory treatment in comparison to the humoral response. On the other hand, it should be noted that due to persistent inflammation in RA, T cells have an exhausted phenotype, which is characterized by the diminished ability to respond to viral antigens (35). Therefore, lower immunogenicity of vaccination in these patients may result not only from immunomodulatory treatment but also from the features of the underlying disease itself.

Although current variants of the SARS-CoV-2 appear to result in a milder course of the disease, COVID-19 still poses a risk to immunocompromised patients. Up to date, vaccination guidelines regarding booster doses for AIIRD patients are based on experts’ opinions, not on well-documented studies. Therefore, scientific evidence to develop optimal booster vaccination regimens for patients treated with immunomodulatory drugs is needed.

The greatest advantage of our study is the assessment of the effect of individual immunomodulatory drugs on both humoral and cellular responses after the COVID-19 booster dose. The biggest limitation of our study is the relatively small sample size and a short period of observation after the booster dose. Studies on a larger group of patients may allow for a more precise determination of booster vaccine immunogenicity in patients treated with specific immunomodulatory drugs, while studies with a longer follow-up period will allow for finding the optimal time between booster doses. Additionally, we did not measure the neutralization capacity of antibodies, and disease flares were based on patient reports. Finally, a limitation on the usefulness of our results is that currently, we do not know the antibody and cellular response levels that can effectively protect against COVID-19 infection in IA patients.

Our study confirmed the necessity and good immunogenicity of COVID-19 booster doses in IA patients. Moreover, we determined the effect of a booster dose on cellular response, which appears to be a sensitive marker for assessing the immunogenicity of COVID-19 vaccination in IA patients. Overall, our results may support health professionals and policymakers on the recommendation for subsequent booster doses to ensure the successful vaccination of immunocompromised patients.

## Data availability statement

The raw data supporting the conclusions of this article will be made available upon reasonable request sent to the corresponding author.

## Ethics statement

The study was reviewed and approved by National Institute of Geriatrics, Rheumatology and Rehabilitation bioethics committee (KBT-3/2/2021). The patients provided their written informed consent to participate in this study.

## Author contributions

Conceptualization, JW, BJ, and MC. Methodology, JW, MM, MC, and EK-W. Validation, JW and EK-W. Formal analysis, JW, MM, MC, and EK-W. Investigation, JW, BJ, LR, AF-G, AK, MP, TB, BL, MM, MC, and EK-W. Resources, BJ, AF-G, BK, WM, and MW. Data curation, JW and BJ. Writing – original draft preparation, JW, MM, MC, and EK-W. Writing – review and editing, BJ, LR, AF-G, KB, AK, MP, TB, BL, BK, WM, and MW. Visualization, MC and EK-W. Supervision, BK, WM, and MW. Project administration, JW and MC. Funding acquisition, KB, BL, and BJ. All authors contributed to the article and approved the submitted version.

## Funding

This work was supported by the National Institute of Geriatrics, Rheumatology and Rehabilitation Statutory Grant (Grant No. S/8, S/9, S/33).

## Conflict of interest

The authors declare that the research was conducted in the absence of any commercial or financial relationships that could be construed as a potential conflict of interest.

## Publisher’s note

All claims expressed in this article are solely those of the authors and do not necessarily represent those of their affiliated organizations, or those of the publisher, the editors and the reviewers. Any product that may be evaluated in this article, or claim that may be made by its manufacturer, is not guaranteed or endorsed by the publisher.

## Supplementary material

Supplementary Figure 1 Representative gating strategy for FACS of PBMC, showing CD3+ lymphocytes with intracellular staining for IFN-γ after viral protein stimulation.

Supplementary Figure 2 Correlation (Spearman’s rank coefficient) between antibody titer before (x-axis) and after (y-axis) booster dose of vaccine in **(A)** healthy controls (HC) group, **(B)** rheumatoid arthritis (RA) group, and **(C)** spondyloarthritis (SpA) group.

Supplementary Figure 3 Kinetics of anti-S-IgG concentration in **(A)** healthy controls (HC) group, **(B)** rheumatoid arthritis (RA) group, and **(C)** spondyloarthritis (SpA) group. In each group, a two-tailed Mann-Whitney U-test was performed. P values were expressed as follows: p<0.0001 as****.

Supplementary Figure 4 Kinetics of viral antigen-stimulated fold change of IFN in **(A)** healthy controls (HC) group, **(B)** rheumatoid arthritis (RA) group, and **(C)** spondyloarthritis (SpA) group. In each group, a two-tailed Mann-Whitney U-test was performed. A p values were expressed as follows: 0.05>p>0.01 as*; p<0.0001 as****.

Supplementary Figure 5 Kinetics of percentage of CD3+ INF-γ+ cells after wild-type viral protein stimulation in **(A)** healthy controls (HC) group, **(B)** rheumatoid arthritis (RA) group, and **(C)** spondyloarthritis (SpA) group. Percentage of CD3+ INF-γ+ cells after delta viral protein stimulation in **(D)** HC group, **(E)** RA group, and **(F)** SpA group before and after a booster dose of COVID-19 vaccine.

## References

[1] Akiyama S Hamdeh S Micic D Sakuraba A. Prevalence and clinical outcomes of COVID-19 in patients with autoimmune diseases: A systematic review and meta-analysis. Ann Rheum Dis (2020) 80:384–91. doi: 10.1136/ANNRHEUMDIS-2020-218946 33051220

[2] Wang Q Liu J Shao R Han X Su C Lu W. Risk and clinical outcomes of COVID-19 in patients with rheumatic diseases compared with the general population: A systematic review and meta-analysis. Rheumatol Int (2021) 41:851–61. doi: 10.1007/S00296-021-04803-9 PMC7941871 33687528

[3] Conway R Grimshaw AA Konig MF Putman M Duarte-García A Tseng LY. SARS–CoV-2 infection and COVID-19 outcomes in rheumatic diseases: A systematic literature review and meta-analysis. Arthritis Rheumatol (2022) 74:766–75. doi: 10.1002/ART.42030/ABSTRACT PMC9011807 34807517

[4] Spiera R Jinich S Jannat-Khah D. Rituximab, but not other antirheumatic therapies, is associated with impaired serological response to SARS- CoV-2 vaccination in patients with rheumatic diseases. Ann Rheum Dis (2021) 80:1357–9. doi: 10.1136/annrheumdis-2021-220604 33975857

[5] Ruddy JA Connolly CM Boyarsky BJ Werbel WA Christopher-Stine L Garonzik-Wang J. High antibody response to two-dose SARS-CoV-2 messenger RNA vaccination in patients with rheumatic and musculoskeletal diseases. Ann Rheum Dis (2021) 80:1351–2. doi: 10.1136/annrheumdis-2021-220656 PMC8843949 34031032

[6] Haberman RH Herati R Simon D Samanovic M Blank RB Tuen M. Methotrexate hampers immunogenicity to BNT162b2 mRNA COVID-19 vaccine in immune-mediated inflammatory disease. Ann Rheum Dis (2021) 80:1339–44. doi: 10.1136/annrheumdis-2021-220597 PMC8219484 34035003

[7] Bugatti S de Stefano L Balduzzi S Greco MI Luvaro T Cassaniti I. Methotrexate and glucocorticoids, but not anticytokine therapy, impair the immunogenicity of a single dose of the BNT162b2 mRNA COVID-19 vaccine in patients with chronic inflammatory arthritis. Ann Rheum Dis (2021) 80:1635–8. doi: 10.1136/annrheumdis-2021-220862 34172502

[8] Braun-Moscovici Y Kaplan M Braun M Markovits D Giryes S Toledano K. Disease activity and humoral response in patients with inflammatory rheumatic diseases after two doses of the pfizer mRNA vaccine against SARS-CoV-2. Ann Rheum Dis (2021) 80:1317–21. doi: 10.1136/annrheumdis-2021-220503 34144967

[9] Furer V Eviatar T Zisman D Peleg H Paran D Levartovsky D. Immunogenicity and safety of the BNT162b2 mRNA COVID-19 vaccine in adult patients with autoimmune inflammatory rheumatic diseases and in the general population: A multicentre study. Ann Rheum Dis (2021) 80:1330–8. doi: 10.1136/annrheumdis-2021-220647 34127481

[10] Deepak P Kim W Paley MA Yang M Carvidi AB Demissie EG. Effect of immunosuppression on the immunogenicity of mRNA vaccines to SARS-CoV-2. Ann Intern Med (2021) 174:1572–85. doi: 10.7326/M21-1757 PMC8407518 34461029

[11] Geisen UM Sümbül M Tran F Berner DK Reid HM Vullriede L. Humoral protection to SARS-CoV2 declines faster in patients on TNF alpha blocking therapies. RMD Open (2021) 7:e002008. doi: 10.1136/rmdopen-2021-002008 34880128 PMC8655347

[12] Seyahi E Bakhdiyarli G Oztas M Kuskucu MA Tok Y Sut N. Antibody response to inactivated COVID-19 vaccine (CoronaVac) in immune-mediated diseases: A controlled study among hospital workers and elderly. Rheumatol Int (2021) 41:1429–40. doi: 10.1007/s00296-021-04910-7 PMC8188953 34109466

[13] Chiang TP-Y Connolly CM Ruddy JA Boyarsky BJ Alejo JL Werbel WA. Antibody response to the Janssen/Johnson & Johnson SARS-CoV-2 vaccine in patients with rheumatic and musculoskeletal diseases. Ann Rheum Dis (2021) 80:1365–6. doi: 10.1136/annrheumdis-2021-221145 PMC8440413 34429320

[14] Prendecki M Clarke C Edwards H McIntyre S Mortimer P Gleeson S. Humoral and T-cell responses to SARS-CoV-2 vaccination in patients receiving immunosuppression. Ann Rheum Dis (2021) 80:1322–9. doi: 10.1136/annrheumdis-2021-220626 PMC8350975 34362747

[15] Ferri C Ursini F Gragnani L Raimondo V Giuggioli D Foti R. Impaired immunogenicity to COVID-19 vaccines in autoimmune systemic diseases. high prevalence of non-response in different patients’ subgroups. J Autoimmun (2021) 125:102744. doi: 10.1016/J.JAUT.2021.102744 34781162 PMC8577991

[16] Sidler D Born A Schietzel S Horn MP Aeberli D Amsler J. Trajectories of humoral and cellular immunity and responses to a third dose of mRNA vaccines against SARS-CoV-2 in patients with a history of anti-CD20 therapy. RMD Open (2022) 8:e002166. doi: 10.1136/RMDOPEN-2021-002166 35361691 PMC8971359

[17] Moyon Q Sterlin D Miyara M Anna F Mathian A Lhote R. BNT162b2 vaccine-induced humoral and cellular responses against SARS-CoV-2 variants in systemic lupus erythematosus. Ann Rheum Dis (2022) 81:575–83. doi: 10.1136/ANNRHEUMDIS-2021-221097 PMC8494536 34607791

[18] Picchianti-Diamanti A Aiello A Laganà B Agrati C Castilletti C Meschi S. ImmunosuppressiveTherapies differently modulate humoral- and T-Cell-Specific responses to COVID-19 mRNA vaccine in rheumatoid arthritis patients. Front Immunol (2021) 12:740249. doi: 10.3389/FIMMU.2021.740249 34594343 PMC8477040

[19] Frey S Chiang TP-Y Connolly CM Teles M Alejo JL Boyarsky BJ. Antibody durability 6 months after two doses of SARS-CoV-2 mRNA vaccines in patients with rheumatic and musculoskeletal disease. Lancet Rheumatol (2022) 4:E241–3. doi: 10.1016/S2665-9913(21)00417-3 PMC8765758 35072108

[20] Farroni C Picchianti-Diamanti A Aiello A Nicastri E Laganà B Agrati C. Kinetics of the b- and T-cell immune responses after 6 months from SARS-CoV-2 mRNA vaccination in patients with rheumatoid arthritis. Front Immunol (2022) 13:846753. doi: 10.3389/FIMMU.2022.846753 35309297 PMC8924958

[21] Benucci M Damiani A Infantino M Manfredi M Grossi V Lari B. Presence of specific T cell response after SARS-CoV-2 vaccination in rheumatoid arthritis patients receiving rituximab. Immunol Res (2021) 69:309–11. doi: 10.1007/s12026-021-09212-5 PMC8319896 34324159

[22] Mrak D Tobudic S Koblischke M Graninger M Radner H Sieghart D. SARS-CoV-2 vaccination in rituximab-treated patients: B cells promote humoral immune responses in the presence of T-cell-mediated immunity. Ann Rheum Dis (2021) 80:1345–50. doi: 10.1136/annrheumdis-2021-220781 34285048

[23] Kamar N Abravanel F Marion O Couat C Izopet J del Bello A. Three doses of an mRNA covid-19 vaccine in solid-organ transplant recipients. New Engl J Med (2021) 385:661–2. doi: 10.1056/NEJMc2108861 PMC8262620 34161700

[24] Connolly CM Teles M Frey S Boyarsky BJ Alejo JL Werbel WA. Booster-dose SARS-CoV-2 vaccination in patients with autoimmune disease: A case series. Ann Rheum Dis (2022) 81:291–3. doi: 10.1136/annrheumdis-2021-221206 PMC11034903 34493492

[25] Assawasaksakul T Sathitratanacheewin S Vichaiwattana P Wanlapakorn N Poovorawan Y Kittanamongkolchai W. Immunogenicity, safety and reactogenicity of a heterogeneous booster following the CoronaVac inactivated SARS-CoV-2 vaccine in patients with SLE: A case series. RMD Open (2021) 7:e002019. doi: 10.1136/rmdopen-2021-002019 34862313 PMC8646968

[26] Curtis JR Johnson SR Anthony DD Arasaratnam RJ Baden LR Bass AR. American College of rheumatology guidance for COVID-19 vaccination in patients with rheumatic and musculoskeletal diseases: Version 4. Arthritis Rheumatol (2022) 74:e21. doi: 10.1002/ART.42109 35474640 PMC9082483

[27] Bieber A Sagy I Novack L Brikman S Abuhasira R Ayalon S. BNT162b2 mRNA COVID-19 vaccine and booster in patients with autoimmune rheumatic diseases: A national cohort study. Ann Rheum Dis (2022) 81:1028–35. doi: 10.1136/ANNRHEUMDIS-2021-221824 35418481

[28] Fragoulis GE Karamanakos A Arida A Bournia VK Evangelatos G Fanouriakis A. Letter: Clinical outcomes of breakthrough COVID-19 after booster vaccination in patients with systemic rheumatic diseases. RMD Open (2022) 8:2279. doi: 10.1136/RMDOPEN-2022-002279 PMC8918070 35246472

[29] Benucci M Damiani A Gobbi FL Lari B Grossi V Infantino M. Role of booster with BNT162b2 mRNA in SARS-CoV-2 vaccination in patients with rheumatoid arthritis. Immunol Res (2022) 70(4):493–500. doi: 10.1007/S12026-022-09283-Y PMC9092040 35543863

[30] Connolly CM Chiang TP-Y Teles M Frey S Alejo JL Massie A. Factors associated with poor antibody response to third-dose SARS-CoV-2 vaccination in patients with rheumatic and musculoskeletal diseases. Lancet Rheumatol (2022) 4:e382–4. doi: 10.1016/S2665-9913(22)00065-0 PMC8963771 35368386

[31] le Moine C Soyfoo MS Mekkaoui L Dahma H Tant L. Waning humoral immunity of SARS-CoV-2 vaccination in a rheumatoid arthritis cohort and the benefits of a vaccine booster dose. Clin Exp Rheumatol (2022). doi: 10.55563/CLINEXPRHEUMATOL/TI3TVU 35699073

[32] Ferri C Gragnani L Raimondo V Visentini M Giuggioli D Lorini S. Absent or suboptimal response to booster dose of COVID-19 vaccine in patients with autoimmune systemic diseases. J Autoimmun (2022) 131:102866. doi: 10.1016/J.JAUT.2022.102866 35841684 PMC9271490

[33] Assawasaksakul T Sathitratanacheewin S Vichaiwattana P Wanlapakorn N Poovorawan Y Avihingsanon Y. Immunogenicity of the third and fourth BNT162b2 mRNA COVID-19 boosters and factors associated with immune response in patients with SLE and rheumatoid arthritis. Lupus Sci Med (2022) 9:e000726. doi: 10.1136/LUPUS-2022-000726 35902168 PMC9340581

[34] Landewé RBM Kroon FPB Alunno A Najm A Bijlsma JW Burmester G-RR. EULAR recommendations for the management and vaccination of people with rheumatic and musculoskeletal diseases in the context of SARS-CoV-2: The November 2021 update. Ann Rheum Dis (2022). doi: 10.1136/ANNRHEUMDIS-2021-222006 35197264

[35] Frenz T Grabski E Buschjäger D Vaas LAI Burgdorf N Schmidt RE. CD4(+) T cells in patients with chronic inflammatory rheumatic disorders show distinct levels of exhaustion. J Allergy Clin Immunol (2016) 138:586–9.e10. doi: 10.1016/J.JACI.2016.04.013 27264455

