# Supplementary figures and images for: Humoral and cellular immunogenicity of COVID-19 booster dose vaccination in inflammatory arthritis patients
Source: Front Immunol. 2022 Oct 31;13:1033804. doi: 10.3389/fimmu.2022.1033804 (PMC9659732; doi:10.3389/fimmu.2022.1033804)

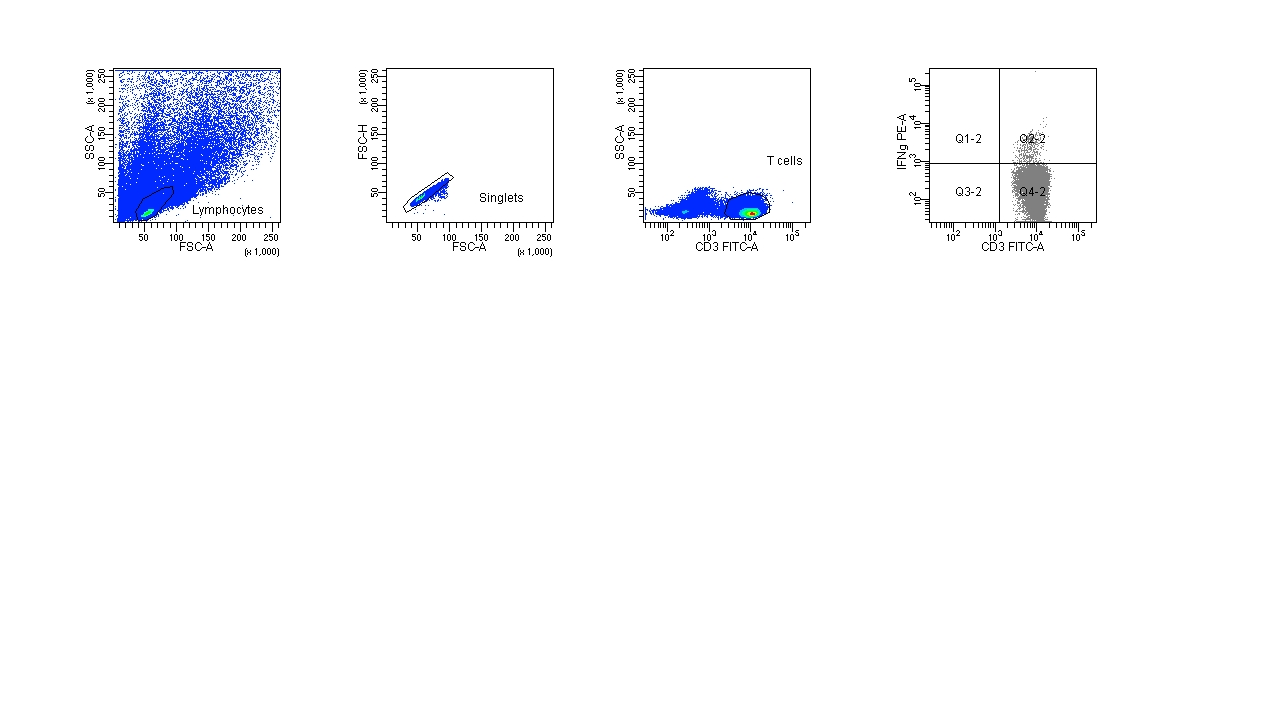

Supplement: Supplementary Figure 1 — Representative gating strategy for FACS of PBMC, showing CD3+ lymphocytes with intracellular staining for IFN-γ after viral protein stimulation. [file Image_1.tif]

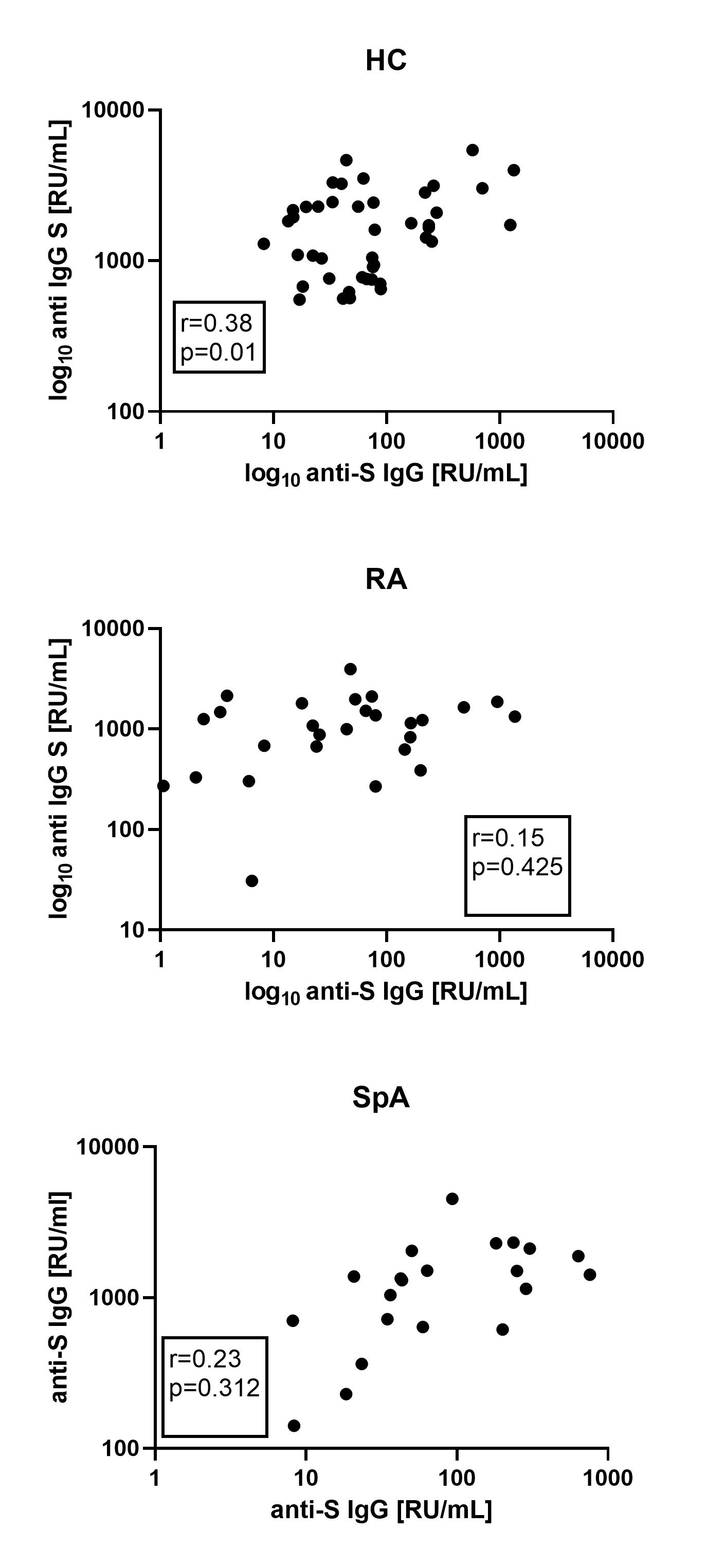

Supplement: Supplementary Figure 2 — Correlation (Spearman’s rank coefficient) between antibody titer before (x-axis) and after (y-axis) booster dose of vaccine in (A) healthy controls (HC) group, (B) rheumatoid arthritis (RA) group, and (C) spondyloarthritis (SpA) group. [file Image_2.tif]

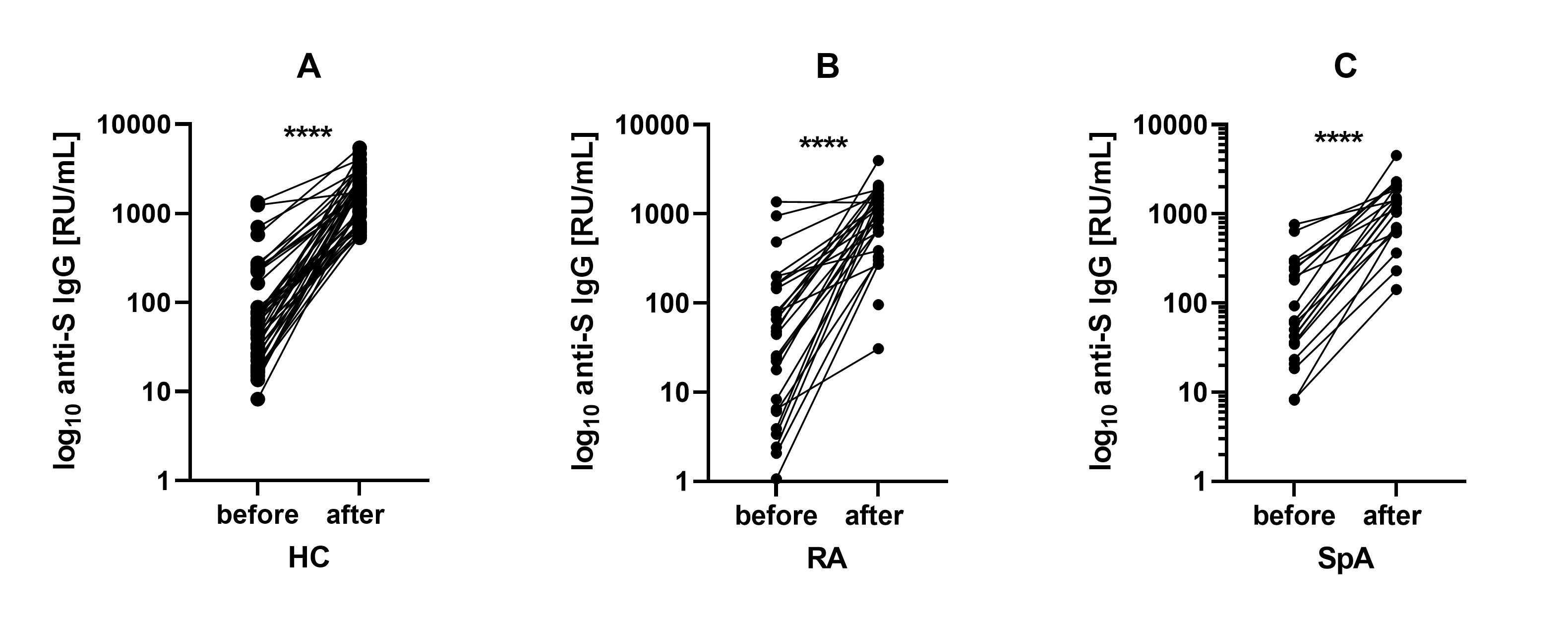

Supplement: Supplementary Figure 3 — Kinetics of anti-S-IgG concentration in (A) healthy controls (HC) group, (B) rheumatoid arthritis (RA) group, and (C) spondyloarthritis (SpA) group. In each group, a two-tailed Mann-Whitney U-test was performed. P values were expressed as follows: p<0.0001 as****. [file Image_3.tif]

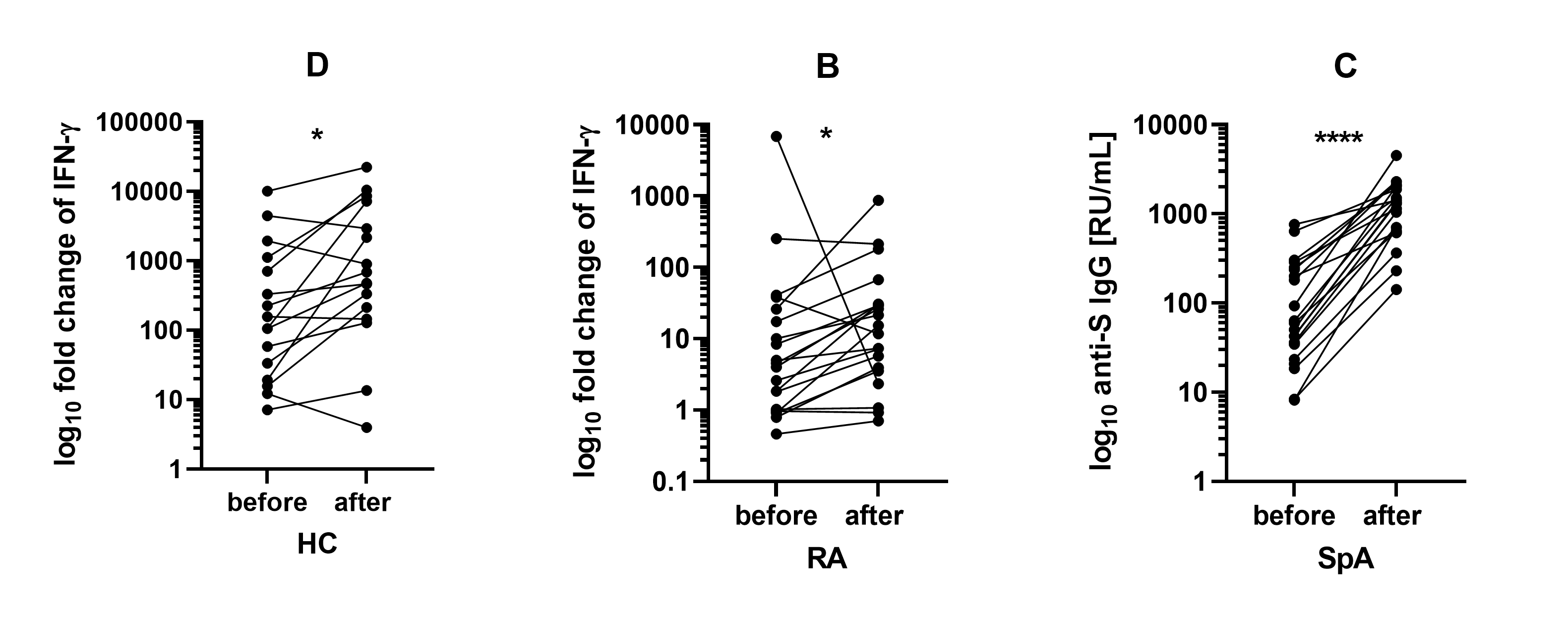

Supplement: Supplementary Figure 4 — Kinetics of viral antigen-stimulated fold change of IFN in (A) healthy controls (HC) group, (B) rheumatoid arthritis (RA) group, and (C) spondyloarthritis (SpA) group. In each group, a two-tailed Mann-Whitney U-test was performed. A p values were expressed as follows: 0.05>p>0.01 as*; p<0.0001 as****. [file Image_4.tif]

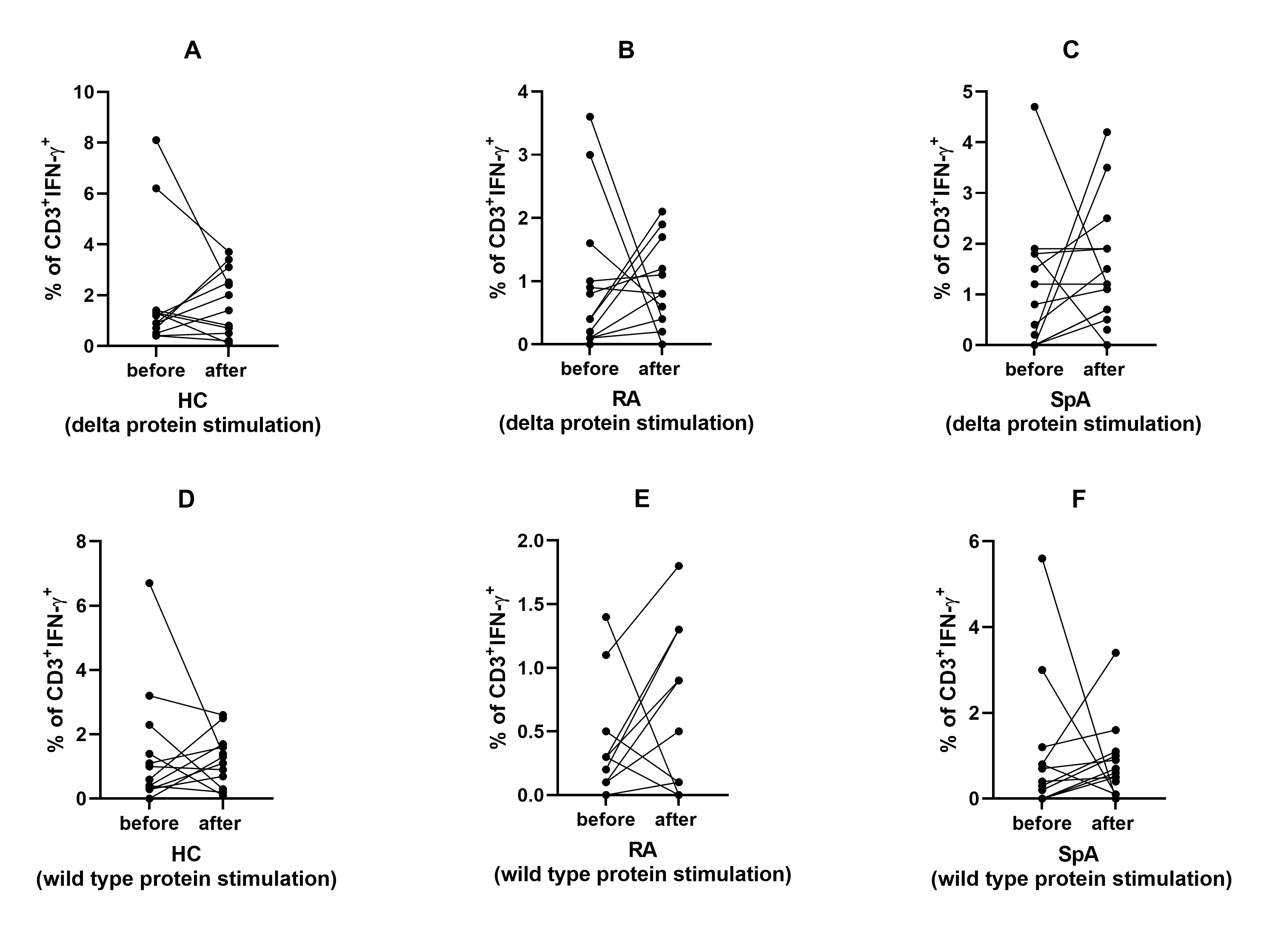

Supplement: Supplementary Figure 5 — Kinetics of percentage of CD3+ INF-γ+ cells after wild-type viral protein stimulation in (A) healthy controls (HC) group, (B) rheumatoid arthritis (RA) group, and (C) spondyloarthritis (SpA) group. Percentage of CD3+ INF-γ+ cells after delta viral protein stimulation in (D) HC group, (E) RA group, and (F) SpA group before and after a booster dose of COVID-19 vaccine. [file Image_5.tif]
